# How Stereotype Threat Influences Cognitive Performance: It All Depends on How You Feel!

**DOI:** 10.5334/irsp.976

**Published:** 2025-02-17

**Authors:** Saša Drače, Verda Dolarević, Elma Šašić

**Affiliations:** 1 University of Sarajevo, Bosnia and Herzegovina

**Keywords:** stereotype threat, mood behavior model, emotions, situational demands, task difficulty, effort, task performance

## Abstract

Studies have shown that mood could be used as diagnostic information for the assessment of situational demands and that, as such, it can regulate resource mobilization. Accordingly, it was found that negative mood causes overestimation of situational demands, which then leads to effort exertion during performance on easy tasks but disengagement on difficult tasks. The present research investigated whether this mood-motivation relation could be extended to specific emotions to explain the effect of stereotype threat (ST). In order to answer this question, the participants in the standard (fear-based) ST and the no-ST conditions had to perform easy (Study 1) or difficult (Study 2) cognitive tasks. To further explore the hypothetical role of threat-related emotions in each study we introduced another condition in which participants under ST were induced to feel anger (i.e., an emotion theoretically characterized by the perception of low situational demands). Although both ST conditions consistently showed greater stereotype-related concerns compared with the control (no-ST) group, the expected increase in easy task performance (Study 1) and decrease in difficult task performance (Study 2) were observed only in the standard (fear-based) ST condition, but not when participants under ST experienced anger. Our findings suggest that specific emotions emerging under ST could govern motivational processes and account for the effect of ST. Accordingly, the way that individuals appraise ST may have an important impact on task performance.

Stereotype threat (ST) refers to the phenomenon whereby individuals perform more poorly on a task when a relevant stereotype or stigmatized social identity is made salient in the performance situation (Schmader & Johns 2003). For instance, studies have shown that African American students performed worse on a standardized test when it was presented as an intelligence assessment, compared to when the identical test was framed as a task unrelated to intelligence; no such differences were observed for White American students (Steele & Aronson 1995). Similar effects have been observed across various domains and groups, affecting women’s performance in math (Spencer, Steele & Quinn 1999) and spatial tasks (Lin et al. 2021), as well as the performance of white men in sports (Stone et al. 1999) and memory performance in the elderly (Armstrong et al. 2017).

Over the years, many studies have examined several mechanisms which underlie this phenomenon (for an overview, see Jamieson & Harkins 2007; Pennington et al. 2016; Schmader, Johns & Forbes 2008; Spencer, Logel & Davies 2016). In the beginning, the assertion that reduced performance results from added pressure or concern (Steele & Aronson 1995) has led many researchers to examine the role of affective mechanisms, such as anxiety or evaluation apprehension. However, this idea received mixed support. Whereas some studies found that anxiety and negative task-related thoughts lead to parallel performance impairments under ST (e.g., Bosson et al. 2004; Cadinu et al. 2005; Ford et al. 2004), other studies failed to find such a link (e.g., Aronson et al. 1999; Spencer et al. 1999; Steele & Aronson 1995; Stone et al. 1999).

Lately, some researchers have proposed that anxiety and stereotype-related concerns may have a more indirect effect on task performance by influencing executive functioning (Rydell, Van loo & Boucher 2014; Schmader & Johns 2003; Schmader, Johns & Forbes 2008). More precisely, Schmader and Johns (2003) argued that ST brings additional demands to the test situation (e.g., anxiety, stereotype-related thoughts), which limits working memory capacity that individuals need to perform a task successfully. Thus, cognitive resources that could be invested in task performance are instead expended on processing the information and emotions resulting from the activation of the negative stereotype. In support of this assumption, these authors conducted a series of studies and found that priming self-relevant negative stereotype reduced working memory capacity, measured via an operation span task, which in turn mediated the detrimental effects of ST on women’s math performance (Schmader & Johns 2003).

In the present paper we propose an alternative perspective of emotional influences in the ST phenomenon. Unlike previous research, which relied exclusively on a cognitive approach, our goal was to examine how anxiety and fear of confirming a negative stereotype about a salient group identity may modulate motivational processes, which govern effort intensity and task performance. In line with this, we propose the Mood Behavior Model (MBM; Gendolla 2000; Gendolla & Brinkmann 2005) as a theoretical framework wherein ST effects may be studied and explained.

Building on the affect-information integration approach (Abele & Petzold 1994), the MBM suggests that individuals use their mood as a valuable piece of information for making task-related judgments and appraisals, which is expected to influence the intensity and persistence of their behavior (Gendolla 2000; Gendolla & Brinkmann 2005). For instance, when individuals encounter challenges in performance contexts and consider questions like ‘How difficult is the task?’ or ‘What level of effort will I need to succeed?’ their moods can skew their responses in a mood-congruent manner. Consequently, those in a negative mood may perceive the task as more difficult and more effort requiring than those in a positive mood, leading to a lower subjective perception of task demand in a positive mood than in a negative mood (e.g., Gendolla, Abele & Krüsken 2001). To make predictions about mood effects on resource mobilization, the MBM integrates the informational influence of mood on demand appraisals with the principles of Motivational Intensity Theory (Brehm & Self 1989). More specifically, this theory posits that individuals strive to avoid wasting energy and will mobilize resources in proportion to their subjective perception of task demand, provided they believe that success is both worthwhile and achievable. Based on these principles, mood and objective task difficulty are expected to produce additive informational effects on the level of demand perception, resulting in a crossover interaction effect between mood and task difficulty on the effort intensity (see Gendolla 2012). Specifically, individuals in a negative mood tend to perceive a lower likelihood of success compared to those in a positive mood, leading to greater subjective demands in a negative mood as opposed to a positive one. Therefore, when the task is objectively easy, individuals in a negative mood will exert more effort than those in a positive mood. However, when faced with an objectively difficult task, individuals in a negative mood are less likely to mobilize resources, as they perceive the additive situational demands as excessively high, resulting in disengagement. In contrast, individuals in a positive mood will mobilize relatively significant resources because they perceive the overall task demand as high but still manageable.

To test this hypothesis Gendolla & Krüsken (2001; 2002) conducted a series of studies in which they manipulated participants’ moods and assessed cardiovascular responses during the subsequent performance on easy and difficult cognitive tasks. As a key finding, the researchers observed stronger systolic blood pressure reactivity—an established indicator of effort intensity (Richter, Gendolla & Wright 2016)—in individuals experiencing a sad mood compared to those in a happy mood during easy tasks. Conversely, when the tasks were difficult, cardiovascular reactivity was greater in a happy mood than in a sad mood.

Aside from global mood effects we propose that the original logic of the MBM could potentially be extended to specific emotions as well. As Frijda (1988, p. 354) put it, ‘emotions exist for the sake of signalling states of the world that have to be responded to, or that no longer need response and action.’ What exactly a given emotion signals can be derived from its underlying appraisal pattern (Ellsworth & Scherer 2003; Ortony, Clore & Collins 1988). For instance, anger and fear are both negative emotions that significantly differ in cognitive appraisals that could be relevant to the assessment of performance context. Compared to fear, anger signals that a situation is highly predictable and under personal control, generally leading to high optimism, positive expectations, and a heightened sense of coping potential (Han, Lerner & Keltner 2007; Hemenover & Zhang 2004; Lerner & Keltner 2001; Lerner & Tiedens 2006). Given that high coping potential reduces the perceived difficulty of a task (e.g., Wright & Dismukes 1995), each emotion may provide different types of information for assessing situational demands, which, depending on objective task difficulty, could determine the intensity of effort invested.

It is important to note that while the MBM primarily predicts the affective influence on the intensity of effort, rather than on performance itself, recent empirical evidence suggests that variations in effort investment can directly impact performance outcomes. For example, Sankaran, Szumowska, and Kossowska (2017) conducted a series of studies across various cognitive tasks, consistently finding that increased self-reported cognitive effort was linked to improved performance. Similarly, Bijleveld, Custers, and Aarts (2012) demonstrated that manipulating effort requirements led to faster performance in a finger-tapping task when higher effort levels were required. Further support comes from research utilizing physiological markers of cognitive effort, relying on pupillometry (for an overview, see van der Wel & van Steenbergen 2018). For instance, studies have shown that greater pupil dilation during the preparatory phase is positively correlated with better performance on inhibitory tasks, such as the Stroop test (Rondeel et al. 2015) and the Anti-saccade task (Unsworth, Miller & Robison 2023; Wang, Brien & Munoz 2015). Consistent results have also been observed in studies examining other cognitive functions where variations in pupil size reflected successful encoding and recall of memories (Kucewicz et al. 2018). Based on these findings, we expected that basic MBM assumptions regarding the informational influence of emotions on resource mobilization may be extended to predict performance outcomes.

This relation between effort and performance is also recognized in the ST literature. According to the mere effort model (Jamieson & Harkins 2007; Jamieson & Harkins 2009), ST motivates individuals to perform well, thereby enhancing the dominant response in a given task. When the task is easy, the dominant response is usually correct, and the added motivation from stereotype threat tends to improve performance (e.g., Ben-Zeev, Fein & Inzlicht, 2005; O’Brien & Crandall 2003). However, when the dominant response is incorrect, which is more common for difficult tasks, performance tends to decline. Importantly, even in these cases, the mere effort model suggests that individuals under ST remain motivated to correct their mistakes, provided they recognize them and have the opportunity to do so. In support of this, Jamieson and Harkins (2007) found that participants taking an anti-saccade test under ST struggled to inhibit their reflexive response to the irrelevant cue but quickly corrected the error and shifted their attention to the actual target.

While the mere effort model highlights that the relationship between ST and performance is mediated by effort, the precise psychological mechanism driving these motivational processes remains unclear. Drawing on the extended version of MBM, Drace, Korlat and Đokić (2020) conducted a series of experimental studies exploring the idea that ST-related emotions (i.e., fear) play a key role in appraising situational demands, which in turn could influence both effort intensity and task performance. More precisely, in one study participants were assigned to the ST and the no-ST conditions and had to perform a series of easy logical problems. To explore the role of threat-related feelings, the authors introduced another ST condition in which participants received a cue pointing to the actual cause of their feelings right before their performance i.e., standard manipulation which was supposed to suppress the informative value of threat-related feelings (see Gasper & Clore 1998; Gendolla & Krüsken 2002; Schwarz & Clore 1983; Tillema, Cervone & Scott 2001). Consistent with the MBM account, ST had positive effect on easy task performance. Importantly, this effect was moderated by the informative potential of one’s current feelings. Although participants in both ST groups were exposed to the same threatening information, only those in the standard ST condition (without a cue) achieved better performance compared to the control (no-ST) group. Using the same design, in a follow-up study the authors extended the test of the MBM account in a context featuring performance on difficult cognitive tasks where the activation of a negative group stereotype led to a typical decrease in performance on the working memory test. However, when participants received a cue that called into question the diagnostic value of threat-related feelings as task-relevant information, this effect completely diminished. Taken together, the findings by Drace et al. (2020) provided converging evidence that threat-related feelings could be used as diagnostic information for the assessment of situational demands and, therefore, could regulate the intensity of cognitive effort during task performance.

The present research was designed to further explore this idea using more rigorous test of the MBM assumptions. This appears particularly important because findings by Drace et al. (2020) are in line with some previous research proposing the alternative perspective of the ST effect. For instance, using conceptually similar design, Ben-Zeev et al. (2005; see also Johns, Schmader & Martens, 2005) asked women to complete math problems described either as a problem-solving task (i.e., no threat) or as a math test (i.e., ST). In a third condition, participants under ST were given the opportunity to attribute the negative arousal presumably triggered by the threat to a benign source (subliminal noise). They found that women under ST performed worse on a difficult, threat-irrelevant task than those in the control group. However, this effect was reduced when women under ST were given the opportunity to attribute the negative arousal, presumably triggered by the threat, to a benign source, rather than to the stereotype or the threatened domain. According to the authors, these results suggest that arousal, particularly how it is attributed, may play an important role in the ST effects. Given that arousal and the subjective feeling of fear occur together as parts of the emotional experience (Dienstbier 1989), it is possible that cue manipulations in Drace et al. (2020) studies could also have prevented the arousal effect, which directly questions the basic assumption of the MBM account regarding informative role of ST-related feelings. To circumvent this problem, we conducted two experimental studies adopting an alternative approach that involved manipulating the informative quality of emotional experiences under ST while keeping corresponding negative arousal constant. One way to achieve this is by exposing participants to the same negative group stereotype and guiding them to interpret the situation in a manner that induces different emotions. Specifically, we relied on the extended MBM suggesting that the experience of specific emotions during the performance of easy and difficult tasks may produce contrasting effects on the appraisal of situational demands and effort mobilization (Lerner & Keltner 2001; Lerner & Tiedens 2006; Wright & Dismukes 1995). Based on this reasoning, we replicated the standard ST and control groups from Drace et al.’s (2020) studies, in which participants had to perform easy (Study 1) or difficult (Study 2) cognitive tasks. Crucially, in each study we introduced an additional ST condition in which participants were induced to feel anger, another high-arousal emotion such as fear (Clobert et al. 2022; Russell 1980). According to the MBM account, the impact of ST is expected to vary based on the informational influence of threat-related emotions on individuals’ assessments of situational demands and objective task difficulty. Although fear and anger are both negative and high-arousal emotions, fear is characterized by the perception of higher situational demands (i.e., the evaluation of higher task difficulty) whereas anger is characterized by low perception of situational demands. Consequently, when the task is easy, fear is anticipated to elicit greater effort exertion compared to anger and conversely in the case of difficult tasks. We thus hypothesized that ST would prompt typical increase in performance on easy tasks (Study 1) and deterioration in performance on difficult tasks (Study 2). However, we anticipate these effects to manifest exclusively under the standard (fear-based) ST condition, and not when participants subjected to ST are induced to experience anger.

## Study 1

In Study 1, participants in both the standard (fear-based) stereotype threat (ST) and no ST conditions were asked to complete a series of easy problems from Raven’s Progressive Matrices, which is generally used as a test of abstract reasoning linked to fluid intelligence (Styles, Raven & Raven 1998). To test the assumptions of the MBM, we introduced an additional condition where participants under ST were induced to experience anger. If individuals rely on their emotions to assess situational demands, then effort intensity and performance should vary based on the specific emotional experience accompanying ST. According to the MBM, the activation of a negative stereotype should lead to the perception of higher situational demands in the standard fear-based condition but not in the anger-based condition. As the task is easy and the overall task demands are expected to justify the investment of resources, we hypothesized that participants in the standard (fear-based) ST condition would exert more effort and thus perform better than those in the anger-based ST and no ST conditions, between which we expected no difference in performance.

### Method

#### Participants

A total of 94 undergraduates from the University of Sarajevo participated in the experiment. They were randomly assigned to the standard ST, the ST-anger, and the no-ST conditions. Three participants who identified themselves as foreign citizens were excluded, leaving a final sample of 91 individuals (82 women, *M*_age_ = 20.29; *SD* = 1.14). A sensitivity analysis conducted with G*Power (Faul et al. 2007) indicated that the sample size was sufficient to detect significant a priori contrast effects of medium to large size with 80% power.

#### Materials and procedure

Participants took part in groups of four, in a large laboratory room (4 m × 6 m). They were seated in front of personal computers and could not interact with each other. At the beginning of each experimental session, participants completed the self-report questionnaire in which they rated to what extent they felt each of 10 separate emotions: *angry, mad, frustrated, unnerved, sad, scared, anxious, worried, nervous, depressed* (1 = *not at all*; 5 = *very much*). For the ST condition, the study was presented as part of a cross-cultural project aimed to explore the validity of cultural beliefs about the lower intellectual abilities of Bosnians relative to other nations from the former Yugoslavia. Accordingly, participants were also told that they will perform standard test of intellectual abilities which was already used in other countries ostensibly involved in the same project. Previous research showed that this manipulation efficiently induces stereotype-related concerns and feelings dominated by fear (Drace et al. 2020). Therefore, this condition was supposed to replicate typical affective characteristics of the standard ST condition (ST-S). Participants in second ST condition received the same instructions but were oriented to appraise situation in a manner that was supposed to induce anger (ST-A). According to the models of emotion elicitation (Horberg, Oveis & Keltner 2011; Weiss, Suckow & Cropanzano 1999), anger is related to an individual’s perception that (s)he or the corresponding ingroup is a victim of injustice caused by someone else. On the other hand, research also suggests that similar appraisals could be responsible for the emergence of anger under ST (Chateignier et al. 2011; Inzlicht & Kang 2010). In line with this idea, participants in the ST-A condition received a short description of the survey involving students from neighboring countries who expressed high agreement regarding the negative stereotype about the intellectual abilities of Bosnians.^1^ Participants in the control condition were told that they are taking part in a pilot study that aims to determine the validity of the cognitive task that will be used in future studies without any reference to the assessment of intelligence (no-ST).

Following the corresponding manipulations, the experimenter demonstrated one practice trial of Raven’s Progressive Matrices. Once participants understood the procedure, they answered the question about subjective difficulty: *How difficult does the following task appear to you?*’ (1 = *not at all*; 5 = *very much*). Then participants proceeded with the main task, which consisted of six easy problems from the series E (see Drace et al. 2020). After the task performance, participants completed a test experience questionnaire in which they rated to what extent they felt each of 10 separate emotions while taking the test.^2^ A composite measure of *anger, fear* and *sadness*^3^ was computed by averaging participants’ responses for the *angry, mad, frustrated, unnerved* items (Cronbach’s alpha = .82), the *scared, anxious, worried, nervous* items (Cronbach’s alpha = .77), and the *sad* and *depressed* items, respectively (*r* = .58, *p* = .001). To determine if the test description was perceived in a manner consistent with ST manipulations, participants also rated the extent to which they agreed with the following statements: *I am concerned that the researcher will judge Bosnians, as a whole, based on my performance on this test and The researcher will think that Bosnians, as a whole, have less intellectual ability if I did not do well on this test*. Responses were recorded using a 5-point scale (1 = *strongly disagree*; 5 = *strongly agree*) and were averaged to create a global index of national group identity threat (*r* = .61, *p* = .001). We also included an independent item that measured the actual salience of a negative stereotype: *To what extent do you agree that there is a belief in the region that Bosnians have lower intellectual abilities compared to the citizens of neighboring countries?* (1 = *strongly disagree*; 5 = *strongly agree*). At the end participants were thanked and debriefed.

### Results

#### Preliminary analyses

Preliminary analyses revealed that participants in the three groups did not significantly differ regarding their pre-existing emotions (all *F*s < 1). These findings suggest that potential confounds related to these differences cannot account for the expected effect of our main manipulation.

#### Task performance

To test our main hypothesis, we conducted one-way ANOVAs with two orthogonal contrasts: a planned comparison testing our model and a contrast testing the remaining variance (i.e., the only contrast that should not be significant if the tested model fits the data). The test for the planned comparison, which opposed the standard (i.e., fear based) ST condition to other two conditions (ST-S = 2, ST-A = –1, no-ST = –1) was significant, *F*(1, 88) = 48.57, *p* = .001, *η^2^* = .35. Importantly, the orthogonal contrast, opposing the ST-A condition to the no-ST condition (ST-S = 0, ST-A = –1, no-ST = 1) was not significant, *F* (1, 88) = 1.65, *p* = .202, *η^2^* = .02. As expected, the participants in the standard ST condition had a higher percentage of correct answers (*M* = 90.93, *SD* = 9.70) than those in the ST-A (*M* = 54.68, *SD* = 23.94) and the no-ST condition (*M* = 61.43, *SD* = 24.12).

#### Situational demands

Regarding the subjective task difficulty, the planned comparison (ST-S = 2, ST-A = –1, no-ST = 1) was also not significant, *F* < 1. The Scheffe *post-hoc* test revealed no significant differences between the three groups (*M_ST-S_* = 1.92, *SD_ST-S_* = .81; *M_ST-A_* = 1.78, *SD_ST-A_* = .78; *M_no-ST_* = 2.05, *SD_no-ST_* = .80), all *p*s > .05.

#### Test experience questionnaire

*Fear*. The planned comparison for the fear measure, which opposed the standard ST condition to both the ST-A and the control condition (ST-S = 2, ST-A = –1, no-ST = –1) was not significant, *F* < 1. Post hoc comparisons with Scheffe test revealed no significant differences between the three conditions (*M_ST-S_* = 2.04, *SD_ST-S_* = .75; *M_ST-A_* = 1.92, *SD_ST-A_* = .83; *M_no-ST_* = 1.92, *SD_no-ST_* = .69).

*Anger*. The planned comparison for the anger measure, which opposed the ST-A condition to the other two conditions (ST-S = –1, ST-A = 2, no-ST = –1), was significant, *F*(1, 88) = 14.81, *p* = .001, *η^2^* = .14, while the orthogonal contrast (ST-S = –1, ST-A = 0, no-ST = 1) was not, *F*(1, 88) = 2.63, *p* = .107, *η^2^* = .03. Consistent with expectations, participants in the ST-A condition reported higher levels of anger during the test (*M* = 1.93, *SD* = 0.64) than those in the standard (*M* = 1.19, *SD* = .38) and the no-ST group (*M* = 1.47, *SD* = 0.87).

*Sadness*. The results revealed no significant differences in the sadness scores between the three conditions (*M_ST-S_* = 1.37, *SD_ST-S_* = .80; *M_ST-A_* = 1.44, *SD_ST-A_* = .82; *M_no-ST_* = 1.38, *SD_no-ST_* = .64), *F* < 1.

*National identity threat*. The planned comparison, which opposed the two ST conditions to the no-ST condition (ST-S = 1, ST-A = 1, no-ST = –2) was significant, *F*(1, 88) = 14.16, *p* = .001, *η^2^* = .14, while the orthogonal contrast (ST-S = 1, ST-A = –1, no-ST = 0) was not, *F* < 1. As expected, participants in the ST conditions expressed greater concern about their performance in terms of their national identity (*M_ST-S_* = 2.73, *SD_ST-S_* = 1.10; *M_ST-A_* = 2.80, *SD_ST-A_* = 0.82) compared with participants in the no-ST condition (*M* = 1.97, *SD* = 0.99).

*Negative stereotype salience*. Again, the planned comparison, which opposed the two ST conditions to the no-ST condition (ST-S = 1, ST-A = 1, no-ST = –2) was significant, *F*(1, 88) = 7.95, *p* = .005, *η^2^* = .08, while the orthogonal contrast (ST-S = 1, ST-A = –1, no-ST = 0) was not, *F* < 1. Thus, consistent with our manipulations, participants in the ST conditions expressed stronger agreement with the existence of regional belief regarding lower intellectual abilities of Bosnians (*M_ST-S_* = 3.60, *SD_ST-S_* = 1.22; *M_ST-A_* = 3.67, *SD_ST-A_* = 1.21) compared with participants in the no-ST condition (*M* = 2.85, *SD* = 1.39).

### Discussion

In line with previous findings (Ben-Zeev et al. 2005; Drace et al. 2020; O’Brien & Crandall 2003), participants under ST achieved better performance, solving a higher percentage of easy problems than those in the control (no-ST) condition. Importantly, consistent with the MBM account, this effect was moderated by the quality of the accompanying emotional state. Although both ST conditions increased greater stereotype-related concerns than the control (no-ST) condition, the typical performance improvement was observed only in the standard, fear-based ST condition, but not when participants under ST experienced anger. While fear and anger are both negative and high-arousal emotions, unlike anger, fear is theoretically characterized by the perception of higher situational demands. As the task was easy and success appeared possible, participants under ST who experienced fear had valid reasons to exert more effort and consequently achieved better performance than the no-ST group.

One could question our findings because we did not find the expected differences in self-reported emotions. While participants in the ST-anger condition reported elevated levels of anger compared to those in the standard (fear based) ST and the no-ST condition, the comparisons across all three conditions regarding the fear measure were less conclusive. These inconsistent findings could be due to the fact that the ST manipulations, which emphasized negative group comparisons, triggered defensive strategies aimed at preserving self-esteem. Consequently, participants experiencing ST may have felt more comfortable overtly expressing their anger rather than fear, as the latter emotion could be seen as a sort of weakness (Bosson, Haymovitz & Pinel 2004).

Our findings could be questioned due to the absence of the expected effects of ST on the perception of situational demands. One possible reason for this discrepancy is that our assessments of subjective difficulty were based on a demonstration item performed by the experimenter, which was much simpler than the main task. As a result, these unexpected findings are more likely due to the low reliability of our measure rather than an actual lack of effect.

Although our main findings are consistent with the MBM account, it should be noted that Study 1 is limited to performance on an easy cognitive task. Therefore, it does not allow us to conclude whether a similar psychological mechanism is responsible for typical decrease in performance observed during more challenging cognitive tasks (i.e., the classical ST effect). This issue was addressed in Study 2.

## Study 2

Study 2 replicated the design of Study 1, requiring participants to perform the operation-span task (OSPAN), a standard measure of working memory capacity (Đokić, Koso-Drljević & Đapo 2018; Unsworth et al. 2005). Using a very similar task, previous research (Schmader & Johns 2003) showed that the manipulation of ST had detrimental effects on participants’ performance. Based on these findings, we posited that the OSPAN could create a prototypical context for examining performance on difficult tasks (see also Drace et al. 2020). In keeping with our theorizing, we expected that the activation of a negative stereotype should lead to the perception of higher situational demands in the standard, fear-based, ST condition but not in the anger condition. Since the task at hand is objectively difficult, the overall task demands should be perceived as too high to justify the investment of resources. Therefore, participants in the standard ST condition were expected to exert less effort due to disengagement and, consequently, achieve lower performance compared to those in the anger-based ST condition and the no ST condition.

### Method

#### Participants

A total of 108 undergraduates from the University of Sarajevo participated in the experiment. They were randomly assigned to the standard ST, the ST-anger, and the no-ST conditions. Four participants who identified themselves as foreign citizens were excluded, leaving a final sample of 104 individuals (85 women, *M*_age_ = 21.12; *SD* = 3.44). Due to a limited pool of participants for Study 2, we sought to maximize our recruitment to achieve at least a sample size comparable to that of Drace et al. (2020, Experiment 3). Sensitivity analysis conducted with G*Power (Faul et al. 2007) indicated that our sample size was sufficient to detect significant *a priori* contrast effects of a medium to large size with 80% power.

#### Materials and procedure

Participants took part in groups of four, in a large laboratory room (4 m × 6 m). At the start of each experimental session, participants completed the same self-report measure of their current emotions. Based on prior research (Clobert et al. 2022; Russell 1980), in Study 1 we considered both fear and anger to be high-arousal emotions without implementing a corresponding manipulation check. To ensure the construct validity of our manipulations, in Study 2, we included four additional items specifically measuring arousal levels (*tense, upset, calm, and relaxed*). The general cover story and the instructions in the ST conditions were the same as in Study 1 with minor modification in the no ST condition in which the task was presented as a working memory test (Drace et al. 2020; Schmader & Johns 2003). Following the corresponding description of the study, each group went through the OSPAN^4^ training upon which participants were asked to answer the question related to the situational demands (i.e., subjective task difficulty). After the main task, participants completed a test experience questionnaire containing the retrospective measures of *anger, fear, arousal* (respectively, Cronbach’s alphas = .82, .81, .86) *and sadness* (*r* = .44, *p* = .001). The questionnaire also included the two identity threat items from the previous study (*r* = .79, *p* = .001) and one item measuring the actual salience of a negative stereotype. At the end participants were thanked and debriefed.

### Results

#### Preliminary analyses

Preliminary analyses revealed that participants in the three groups did not significantly differ regarding their pre-existing emotions and arousal (all *F*s < 1). These findings suggest that potential confounds related to these differences cannot account for the expected effect of our main manipulation.

#### Task performance

In line with past research (Đokić et al. 2018; Drace et al. 2020; Schmader & Johns 2003), OSPAN performance was operationalized as the absolute span score. The test for the planned comparison, which opposed the standard (i.e., fear-based) ST condition to other two conditions (ST-S = 2, ST-A = –1, no-ST = –1) was significant, *F*(1, 101) = 4.03, *p* = .047, *η^2^* = .04. Importantly, the orthogonal contrast, opposing the ST-A condition to the no-ST condition (ST-S = 0, ST-A = –1, no-ST = 1) was not significant, *F* < 1. As expected, the participants in the standard ST condition had lower span score (*M* = 28.46, *SD* = 16.76) than those in the ST-A (*M* = 35.37, *SD* = 16.54) and the no-ST condition (*M* = 35.42, *SD* = 15.29).

#### Situational demands

The planned comparison (ST-S = 2, ST-A = –1, no-ST = 1) was significant, *F*(1, 101) = 3.96, *p* = .049, *η^2^* = .04. while the orthogonal contrast (ST-S = 0, ST-A = –1, no-ST = 1) was not, *F* < 1. As expected, the participants in the standard ST condition perceived higher subjective task difficulty (*M* = 2.84, *SD* = 1.01) compared to the ST-anger (*M* = 2.40, *SD* = 1.00) and the no ST (*M* = 2.42, *SD* = 0.98) conditions.

#### Test experience questionnaire

*Fear*. The planned comparison for the fear measure, which opposed the standard ST condition to both the ST-A and the control condition (ST-S = 2, ST-A = –1, no-ST = –1) was not significant, *F*(1, 101) = 2.12, *p* = .148, *η^2^* = .01. Post hoc comparisons with Scheffe test revealed no significant differences between the three conditions (*M_ST-S_* = 2.64, *SD_ST-S_* = 1.05; *M_ST-A_* = 2.49, *SD_ST-A_* = .91; *M_no-ST_* = 2.16, *SD_no-ST_* = 1.02).

*Anger*. The planned comparison for the anger measure, which opposed the ST-A condition to the other two conditions (ST-S = –1, ST-A = 2, no-ST = –1), was not significant. *F* < 1. Post hoc comparisons with Scheffe test revealed no significant differences between the three conditions (*M_ST-S_* = 1.72, *SD_ST-S_* = .92; *M_ST-A_* = 1.87, *SD_ST-A_* = .93; *M_no-ST_* = 1.73, *SD_no-ST_* = .82).

*Sadness*. The results revealed no significant differences in the sadness scores between the three conditions (*M_ST-S_* = 1.31, *SD_ST-S_* = .56; *M_ST-A_* = 1.29, *SD_ST-A_* = .57; *M_no-ST_* = 1.40, *SD_no-ST_* = .78), *F* < 1.

*Arousal*. Regarding the measure of arousal, the planned comparison, which opposed the two ST conditions to the no-ST condition (ST-S = 1, ST-A = 1, no-ST = –2) was significant *F*(1, 101) = 4.63, *p* = .033, *η^2^* = .04 while the orthogonal contrast (ST-S = 1, ST-A = –1, no-ST = 0) was not, *F* < 1. As expected, participants in the ST conditions reported higher levels of arousal (*M_ST-S_* = 3.28, *SD_ST-S_* = 1.10; *M_ST-A_* = 3.18, *SD_ST-A_* = 1.13) compared with participants in the no-ST condition (*M* = 2.76, *SD* = 1.08).

*National identity threat*. The planned comparison, which opposed the two ST conditions to the no-ST condition (ST-S = 1, ST-A = 1, no-ST = –2) was significant, *F*(1, 98) = 17.80, *p* = .001, *η^2^* = .15, while the orthogonal contrast (ST-S = 1, ST-A = –1, no-ST = 0) was not, *F* < 1. As expected, participants in the ST conditions expressed greater concern about their performance in terms of their national identity (*M_ST-S_* = 2.53, *SD_ST-S_* = 1.47; *M_ST-A_* = 2.34, *SD_ST-A_* = 1.50) compared with participants in the no-ST condition (*M* = 1.41, *SD* = 0.62).

*Negative stereotype salience*. Again, the planned comparison, which opposed the two ST conditions to the no-ST condition (ST-S = 1, ST-A = 1, no-ST = –2) was significant, *F*(1, 98) = 6.42, *p* = .012, *η^2^* = .06, while the orthogonal contrast (ST-S = 1, ST-A = –1, no-ST = 0) was not, *F* < 1. Thus, consistent with our manipulations, participants in the ST conditions expressed stronger agreement with the existence of regional belief regarding lower intellectual abilities of Bosnians (*M_ST-S_* = 3.53, *SD_ST-S_* = 1.36; *M_ST-A_* = 3.42, *SD_ST-A_* = 1.36) compared with participants in the no-ST condition (*M* = 2.81, *SD* = 1.20).

### Discussion

In line with past research, participants completing OSPAN under ST showed reduced cognitive capacity (Drace et al. 2020; Schmader & Johns 2003). However, consistent with our expectations, this effect was again moderated by the quality of the accompanying emotional state. Although participants in both ST conditions expressed similar elevation in arousal and greater stereotype-related concerns than the control (no-ST) group, the typical decrease in performance was observed only in the standard, fear-based ST condition. Conversely, when participants were induced to experience an emotion theoretically related to low situational demands, ST led to performance similar to that of the no-ST group. In line with this assumption, participants in the standard ST condition perceived higher subjective task difficulty compared to the ST-anger and the no ST conditions. These findings provide further converging evidence that the informative potential of threat-related feelings plays a crucial role in the regulation of cognitive effort task performance. Taken together, Study 2 extended the results of Study 1 showing that emotionally driven motivational processes can also account for the occurrence of the classical ST effect.

One could question our findings because we didn’t observe differences in fear and anger among the three conditions. This issue will be addressed in General Discussion.

## General Discussion

The present research aimed to test an emotional explanation of the ST effect, relying on the MBM (Gendolla 2000; Gendolla & Brinkmann 2005). Consistent with past research (Drace et al. 2020; O’Brien & Crandall 2003), Study 1 showed that participants under ST achieved better performance than those in the control (no-ST) condition. Importantly, this effect, consistent with the MBM assumptions, was moderated by the quality of threat-related emotional state. Although both ST conditions produced greater stereotype-related concerns than the control (no-ST) condition, the typical performance improvement was observed only in the standard, fear-based ST condition, but not when participants under ST experienced anger. Using the same design, Study 2 extended the test of the MBM account in a context featuring performance on difficult cognitive tasks. In line with past research (e.g., Drace et al. 2020; Schmader & Johns 2003), the activation of negative stereotype led to a typical decrease in cognitive performance (i.e., classical ST effect). However, when participants under ST were induced to feel angry, this effect was completely annulled. In addition, Study 2 clearly showed that both ST manipulations had a similar impact on stereotype-related concerns and the elevation of arousal (Blascovich et al. 2001). Consequently, it is unlikely to assume that the anger manipulation produced zero effects on task performance due to potential differences in levels of arousal. Thus, using a different methodological approach, we extended the research by Drace et al. (2020), suggesting that the informative value of specific emotions emerging under ST, rather than an individual’s construal of the meaning of a negative arousal, may modulate motivational processes governing effort intensity and task performance. More importantly, our findings are at odds with the mainstream alternative theoretical explanations. For instance, according to the working memory model (Schmader & Johns 2003), the activation of a negative stereotype should systematically cause a reduction in working memory capacity, which in case of easy cognitive tasks should lead to a lower or, at best, similar performance as in the control (no-ST) group. However, consistent with the MBM, we observed the opposite pattern of results with clear-cut performance improvement, particularly in the standard, fear-based ST condition. One could argue that the observed difference between the two ST conditions could be due to the fact that participants in the anger-based ST condition were more offended (due to some disrespectful statements toward their group) and consequently disengaged from the study, independent of the informative role of emotions in the assessment of situational demands. If this was true, then similar disengagement should be observed during the performance on difficult tasks. However, this hypothesis was disconfirmed by the results of Study 2, where the classic ST effect occurred only in the fear-based condition. Similarly, according to the mere effort model (Jamieson & Harkins 2007; Jamieson & Harkins 2009), ST should motivate participants to perform well, by stimulating prepotent (i.e., dominant) response, which for the easy task is most likely the correct and for the difficult tasks most likely the incorrect answer. As a result, participants under ST should systematically achieve better performance on easy tasks (and conversely lower performance on difficult task). However, this hypothesis was again disconfirmed by the results of the anger-based ST condition of Study 1 and Study 2.

Taken together, our findings yield several theoretical implications. Specifically, by replicating prior findings we have once again demonstrated that ST could lead to a better cognitive performance (Ben-Zeev et al. 2005; Drace et al. 2020; O’Brien & Crandall 2003). Furthermore, our research suggests that this effect could be modulated by the informative influence of threat-related feelings on effort intensity. Consequently, the way individuals appraise ST could play a crucial role in task performance. Thus, when the salience of a negative stereotype evokes emotions associated with high situational demands, such as fear, individuals are likely to exert greater effort and perform better on easy tasks. However, this same emotional response can lead to disengagement and poorer performance on difficult tasks. Conversely, if the same situation is appraised in a manner that activates emotions linked to low situational demands, such as anger, the typical effects of ST are expected to be diminished. Accordingly, our findings also contribute to the existing literature on the MBM (Gendolla 2000; Gendolla & Brinkmann, 2005). Research originally conducted on the MBM has only compared the effects of a positive versus negative mood. Thus, our study joins recent empirical demonstrations showing that basic principles of the MBM could potentially be extended to the influence of specific emotions as well (Drace et al. 2020; see also Wang, Pegna & Framorando 2023).

### Limitations and Directions for the Future Research

The present study has a number of strengths, but some limitations are worth noting. The first one concerns our difficulty in consistently capturing the expected emotional experience under ST. As we have already pointed out, one reason for these results could be the temporary activation of self-enhancement motives, which might particularly interfere with self-reports of fear (e.g., Bosson et al. 2004). Another reason could be that emotions were assessed retrospectively after the task performance, when the effect of ST manipulations might have dissipated (e.g., Robinson & Clore 2002; Sigall & Mills 1998). This could explain why, in Study 2, we failed to capture the expected emotional changes in the anger condition. Yet, as suggested by some authors (e.g., Fayant et al. 2017), when the results on the main dependent variable are in line with predictions and there is no plausible alternative explanation, manipulation checks should not play an important role. Therefore, the clear and consistent difference in task performance between the anger-based and fear-based ST conditions cannot be obscured by the inconsistent effects on the emotion self-reports. Future research could address this issue by including indirect measures, such as coding of non-verbal behavior (e.g., Bosson et al. 2004) or recording physiological reactions, which may provide more accurate insights into people’s emotional states (e.g., Bradley et al. 1990; Brownley et al. 2000), thereby bypassing the problems associated with self-reports.

Our study is limited by the lack of information regarding actual effort, which in the present case was not directly measured but rather inferred from the performance. Consequently, we provide only partial evidence supporting the MBM account. This could be an issue because the relation between mobilization of resources and behavior is not always direct, that is, increased effort does not consistently lead to improved performance (Gendolla & Richter 2010). Therefore, future research should include valid measures of mental effort, such as eye-tracking or physiological indicators (see Richter et al. 2016; van der Wel & van Steenbergen 2018), to directly explore the underlying processes of the ST effects.

Finally, in the present study, we utilized easy and difficult tasks that engage different cognitive processes (fluid intelligence vs. working memory). Adopting a more integrated approach, using tasks of varying difficulty within the same cognitive context, could offer a clearer and more consistent comparison of how the interaction between ST and task difficulty influences performance.

## Conclusion

The aim of this research was to investigate the motivational mechanisms of affective influence in the ST phenomenon. Consistent with the assumptions of the MBM and prior research by Drace et al. (2020), our findings provide further evidence that feelings experienced under ST can serve as diagnostic indicators for assessing situational demands and consequently, can regulate the exertion of effort during task performance. Accordingly, the way individuals appraise a negative group stereotype could play a determinant role in cognitive achievement.

## Data Accessibility Statement

All data and materials can be accessed on this page: https://osf.io/enhyk/?view_only=554bccc376504ae889c84a74180acc74.

## Footnotes

The instructions are available on this page: https://osf.io/enhyk/?view_only=554bccc376504ae889c84a74180acc74

Given that the assessment of emotions before the main task might bring attention to the true cause of participants’ current feelings, potentially rendering them irrelevant for assessing situational demands, the retrospective self-reports appeared to be the best alternative to ensure the manipulation check without undermining the testing of our main hypothesis.

Previous research has indicated that anger inductions can result in mixed feelings, accompanied by an increase in sadness (Drace & Efendic, 2022). Since sadness is theoretically linked to low situational demands, similar to fear, our aim was to ensure that our anger manipulation properly induced the corresponding affective state.

A detailed description of the OSPAN can be found on this page: https://osf.io/hkrg9.
